# Differentiation between normal and metastatic lymph nodes in patients with skin melanoma: Preliminary findings using a DIXON-based whole-body MRI approach

**DOI:** 10.1016/j.ejro.2024.100560

**Published:** 2024-03-19

**Authors:** C. Brussaard, L. Faggioni, F.E. Ramirez-Barbosa, M. Vervoort, Y. Jansen, B. Neyns, J. de Mey, I. Willekens, D. Cioni, E. Neri

**Affiliations:** a Universitair Ziekenhuis Brussel, Department of Radiology, Belgium; b Academic Radiology, Department of Translational Research and of New Surgical and Medical Technologies, University of Pisa, Italy; c Universitair Ziekenhuis Brussel, Department of Biotechnology, Belgium; d Universitair Ziekenhuis Brussel, Department of Medical Oncology, Belgium; e Academic Radiology, Department of Surgical, Medical and Molecular Pathology and Critical Care Medicine, University of Pisa, Italy

**Keywords:** Metastatic melanoma, Lymph nodes, Magnetic resonance whole body imaging, DIXON sequence

## Abstract

**Purpose:**

Metastatic melanoma lymph nodes (MMLns) might be challenging to detect on MR-WBI, as both MMLns and normal lymph nodes (NLns) can show restricted water diffusion. Our purpose is to assess the potential contribution of the DIXON sequence in differentiating MMLns from NLns.

**Material and methods:**

We followed a cohort of 107 patients with stage IIIb/c and IV skin melanoma for 32 months using MR-WBI with DIXON, STIR, and DWI/ADC sequences. We compared signal intensity (SI) values of MMLns and NLns in the four series of the DIXON sequence (in/out-of-phase, fat_only, and water_only series). The fat fraction (SI_fat_only_/SI_in_) and the long:short axis ratio of MMLns were calculated. The fat fraction was also calculated in the fatty hila of NLns.

**Results:**

All MMLns (8 from 7 patients) showed SI_out_>SI_in_ with a mean fat fraction of 10%. In 40 normal fatty hila (25 patients), the proportion of SI_out_>SI_in_ was 100% and mean fat fraction was 89% (p<0.001 for fat fraction, Mann-Whitney U-test). In the cortex of NLns, a SI_out_>SI_in_ pattern was identified in 41/113 cases from 19/40 patients. The median long:short axis ratio in MMLns was 1.13 (range 1.03–1.25).

**Conclusion:**

The combination of three features of MMLns (SI_out_>SI_in_, low-fat fraction and rounded shape) might hold promise in differentiating NLns from MMLns in patients with skin melanoma. Further research is warranted due to the small number of MMLns in our cohort.

## Highlights

• MR-WBI-based surveillance for melanoma lymph node metastases typically includes three sequences (DIXON, STIR and DWI).
• DIXON could improve upon MR-WBI performance in differentiating metastatic melanoma (MMLns) from normal (NLns) lymph nodes.
• On DIXON images, MMLns showed a drop-in effect (SI out-of-phase > SI in-phase) in 100% of cases.
• Our preliminary findings suggest that the DIXON sequence may play a role in early MMLns detection.

## 1 Introduction

The prognosis of patients with malignant skin melanoma depends on the characteristics of the primary tumor and on the timely detection of metastases, which may spread through a hematogenous and/or lymphatic route. Two-thirds of melanoma metastases can be found in the drainage area of regional lymph nodes and may present as satellite lesions (located at a distance less than 2 cm from the primary tumor), in-transit lesions (between 2 cm and the first draining node), micrometastases (in the sentinel node, too small to detect by clinical examination), and macrometastases (clinically detectable or detected on medical imaging) [1].

Although magnetic resonance whole body imaging (MR-WBI) has not been established in the 2019 Cochrane Review as a recommended image modality for the screening and follow-up of malignant melanoma metastases [2], MR-WBI including diffusion-weighted imaging (DWI) is being increasingly used in this setting [3]. However, while DWI can generally be advantageous in detecting metastatic lesions in organs, it may be limited in distinguishing normal lymph nodes (NLns) from metastatic melanoma lymph nodes (MMLns), since both might exhibit water diffusion restriction [3].

We hypothesized that owing to its multiparametric nature, the DIXON sequence could provide additional information that could aid in the differential diagnosis by revealing differences in morphological and signal intensity properties. In particular, we postulated that the paramagnetic behavior of melanin inside MMLns could lead to signal changes detectable on DIXON images. The first report of the paramagnetic properties of melanin in cultured melanoma cells in vivo, resulting in a high signal intensity (SI) on T1-weighted images, dates back to as early as 1997 [4]. However, to our knowledge the effect of melanin on DIXON images has not been systematically investigated so far.

Our purpose was to obtain minable data from the DIXON sequence, based on histology- or PET/CT-proven NLns and MMLns as ground truth, that should improve radiologists’ performance in differentiating NLns from MMLns.

## 2 Materials and methods

### 2.1 Patient population

The ethical committee of our hospital approved this study (ClinicalTrials.gov Identifier: NTC02907827). All procedures complied with relevant laws and institutional guidelines, and written informed consent was obtained from all patients.

From November 2014 to November 2019, we collected a cohort of 107 patients with histologically proven primary cutaneous melanoma. All patients (men/women 52/55, mean age 58 years, range 28–99 years) were disease-free following resection of macro-metastases, or had a durable complete response (CR) or partial response (PR) following systemic therapy, resulting in a stable clinical status for at least three years [3]. Patients underwent an MR-WBI examination every four months as part of their oncological surveillance program. In seven patients, eight MMLns were found (histologically proven or visible on subsequent PET/CT imaging). The fatty hilum of 40 NLns from 25 patients and the cortex of 113 NLns in 40 patients of this cohort were also examined. The follow-up of the clinical records was extended to June 2021 to ensure that NLns had been identified correctly.

We excluded patients with known disturbances of iron hemostasis (as per medical records), to avoid any bias due to SI changes in the DIXON series as a result of iron deposition inside lymph nodes [5], [6], [7].

### 2.2 Imaging protocols

All MRI examinations were carried out on a commercial 3.0-Tesla scanner (MAGNETON Skyra, Siemens Healthcare, Erlangen, Germany). The MR-WBI protocol for the surveillance of malignant melanoma included an axial 3D T1-weighted 2-point DIXON-VIBE sequence, a coronal STIR sequence, and an axial DWI sequence spanning the entire body from the skull to the feet. The parameters of the axial DIXON sequence were the following: repetition time 4 ms, echo time 1.2 ms: 2.4 ms (out-of-phase: in-phase), flip angle 9°, voxel size 1.2 ×1.2 ×3.0 mm, field-of-view 460 ×302 mm. The axial DWI series was acquired with b-values of 50 s/mm² and 800 s/mm², followed by ADC images calculated from the aforementioned DWI series [8].

### 2.3 Image evaluation

To record the shape and dimensions of all MMLns, we measured their shortest diameter in the axial plane and calculated the ratio between the longest and shortest axes. We also evaluated the presence of a complete black boundary (India ink) artifact surrounding the lesion in out-of-phase images, and we compared literature data on NLns [ultrasonography (US) for normal peripheral lymph nodes, CT/MRI for mediastinal and abdominal ones] with the morphological findings from MMLns (i.e., cortex-hilum differentiation, shape, short axis, long:short axis ratio, border delineation on out-of-phase images).

To evaluate lymph node SI, we measured the SI of the fatty hilum of NLns of the axillary and inguinal regions in the out-of-phase, in-phase, fat_only, and water_only series. To this purpose, we placed a free-hand region of interest (ROI) in the water_only series, and duplicated it by copy-and-paste in the other three series of the DIXON sequence, taking care to excluding the black boundary artifact in out-of-phase images.

Due to the very small cortical thickness of NLns, we placed pixel ROIs in the NLns cortex on the water_only images of the DIXON sequence (where the cortex was most visible), and copied and pasted them on the corresponding fat_only images. On the latter, we repositioned the pixel ROIs in the neighborhood of an area where SI was lower than 15 arbitrary units (reference position with an arbitrary limit value), as the NLns cortex has a low-lipid content. The reference pixel ROI was replicated in the remaining series of the DIXON sequences under visual control of the corresponding out-of-phase series, so as to avoid the black boundary artifact. SI values were averaged out, and the procedure was repeated for MMLns.

### 2.4 Statistical analysis

In our series, only 8 MMLns were detected in 7 patients. While this did not prevent a formal statistical analysis of quantitative data, the very small size of the MMLns cohort and the large imbalance between the NLns and MMLns groups suggest that the results of such analysis should be regarded with caution, and our findings should be seen as no more than preliminary, needing confirmation on a larger patient sample (ideally from multicenter trials).

Fat fractions were derived for hilum ROIs and MMLns (mean SI_fat_only_/mean SI_in_), and SI_out_ values were compared to SI_in_ values inside the same ROIs. Continuous variables were expressed as median and range, and fat fractions as percentages. The two-tailed Mann-Whitney U-test was used for group comparisons, with a p-value less than 0.05 being set as threshold for statistical significance.

## 3 Results

All patients with the 8 MMLns and all those with NLns as the control group met the inclusion criteria.

### 3.1 Morphology

The most detailed reports on the morphology of superficial NLns are based on US, which can provide the best spatial and contrast resolution for this task compared to CT and MRI [9], [10], [11], [12], [13]. For neck NLns, a lack of differentiation between cortex and fatty hilum has been described, with NLns in levels Ib and II having an upper short axis limit of 8 mm, whereas this limit for NLns in other neck levels is 5 mm. NLns with a short axis up to 5 mm can be rounded, while those larger than 5 mm are typically bean-shaped.

The short axis of axillary, inguinal, and mediastinal NLns should not exceed 10 mm [9], [10], except for subcarinal NLns, which can be as large as 12–15 mm in short axis [11], [12]. The short axis limits of abdominal NLns can vary from 6 mm for retrocrural NLns to 8 mm and 10 mm for pelvic and retroperitoneal ones, respectively.

**Fig. 1:**
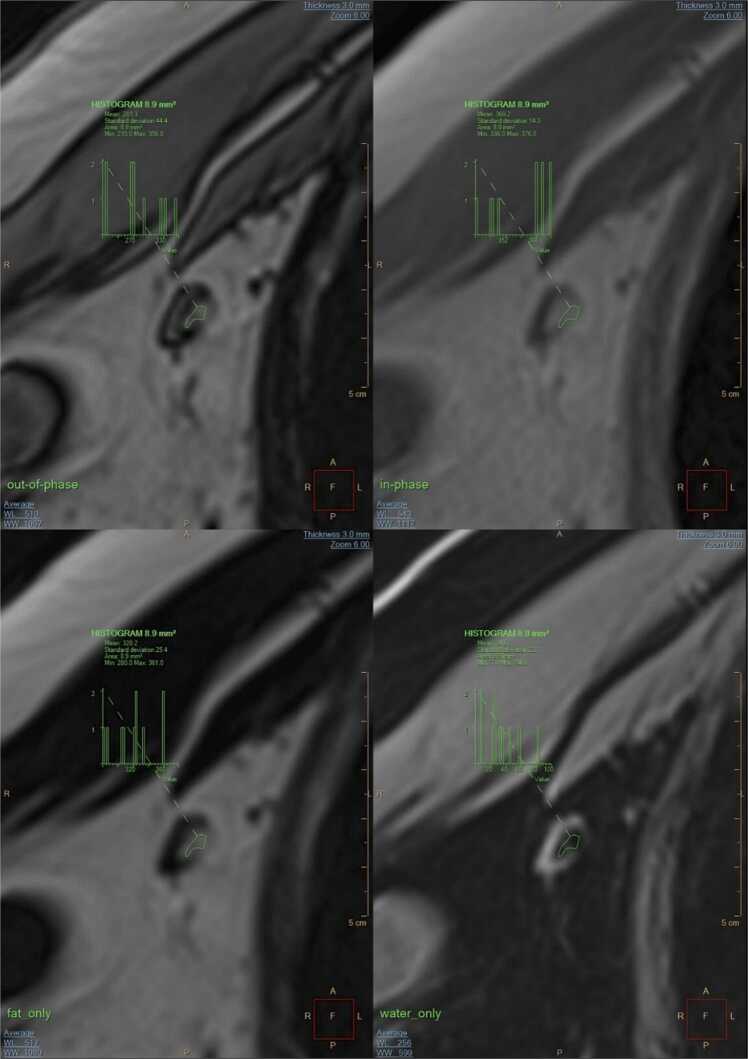
Illustration of a NLn in the out-of-phase, in-phase, fat_only, and water_only series derived from the DIXON sequence. The ROI was replicated in each series by copy-and-paste. The SI_in_>SI_out_ in the fatty hilum reflects the drop-out effect. The ROI has different locations in the hilum on out-of-phase and in-phase images due to misregistration of protons from water and fat molecules, which hampers the generation of exact maps (e.g., SI_out_ - SI_in_). In the out-of-phase series, the cortex is surrounded by a black boundary artifact on the outside and the inner border between the cortex and fatty hilum. The cortex is best highlighted in the water_only series.

While cortex-hilum differentiation can be quite clear for axillary and inguinal NLns, it is often less evident for mediastinal and abdominal NLns. The cortical rim typically has a width of up to 4 mm for axillary NLns and 2.5 mm for inguinal NLns, with a long:short axis ratio between 1.5 and 2 [13]. All NLns should be sharply demarcated from the surrounding extranodal fat (Fig. 1).

**Fig. 2:**
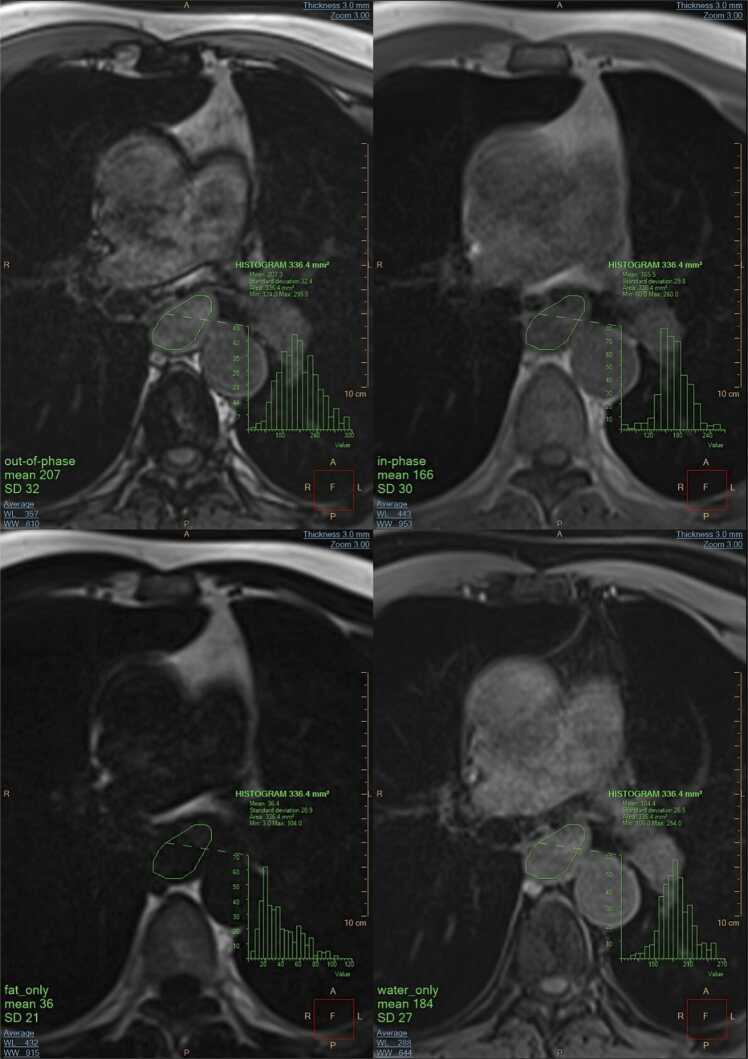
Illustration of a subcarinal MMLn in the out-of-phase, in-phase, fat_only, and water_only series derived from the DIXON sequence. The ROI was replicated in each series by copy/paste and fitted well in the pathologic lymph node in each series due to its low fat content, resulting in a lower misregistration of fat and water protons compared to a NLn. In the out-of-phase series, the cortex is only surrounded by a black boundary artifact as far as it is surrounded by fat (from 5 to 7 o’clock). The SI_out_>SI_in_ reflects a drop-in effect, as seen in all MMLns examined in this study.

In our cohort, MMLns had a peripheral (3/8, of which one in the occipital region), subcarinal (2/8), hilar (1/8), and mesenteric (2/8) location. All MMLns lacked differentiation between the cortex and hilum and were approximately rounded in shape, with a median long:short axis ratio in the axial plane of 1.13 (range 1.03–1.25). The short axis ranged from 7 mm to 25 mm, and 7 out of 8 MMLns had a short axis exceeding 10 mm. The nodal borders on the water_only images were irregularly lobulated in 7 of 8 MMLns. The black boundary artifact was only visible when the nodes were entirely surrounded by fat (5/8 MMLns) (Fig. 2).

### 3.2 Lymph node SI features

**Fig. 3:**
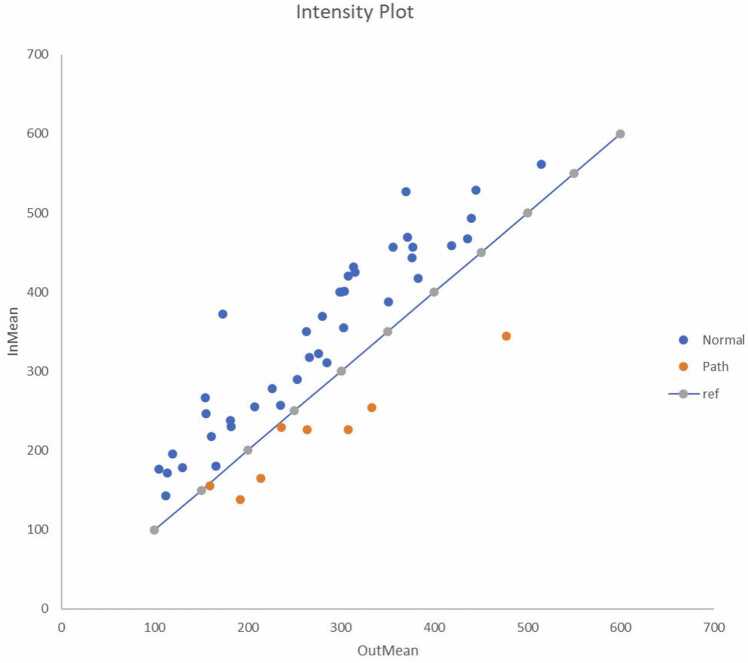
The *x*-axis represents the mean SI of the out-of-phase series of the DIXON sequence, and the *y*-axis is the mean SI of the in-phase series. The reference line represents the position where SI_out_ equals SI_in_. The blue dots express the SI of fatty hila. In the latter, SI_in_ > SI_out_ and all the blue dots were above the reference line due to the drop-out effect. Orange dots express the SI of the MMLns (SI_out_ > SI_in_), and were below the reference line due to the drop-in effect.

**Fig. 4:**
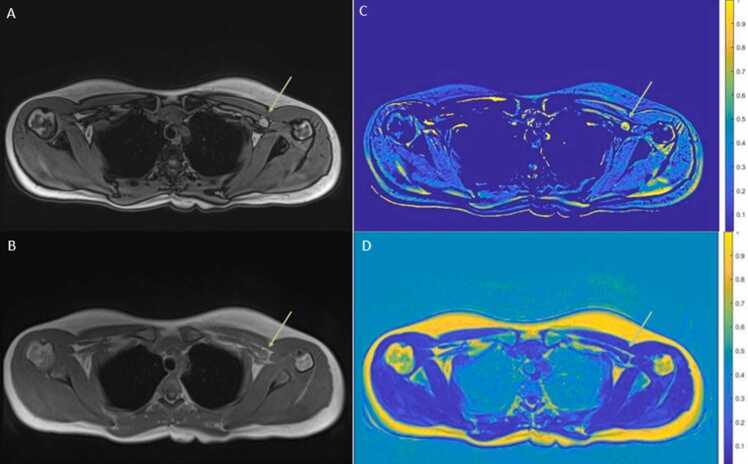
Images A and B represent out-of-phase (A) and in-phase (B) images. Image C is the map prototype calculated as SI_out_ - SI_in_ (noise reduction and image enhancement). Image D shows the fat-fraction map calculated as SI_fat_only_ / SI_in-phase_. In a MMLn (arrow) a visually higher SI was observed in the out-of-phase image compared to the in-phase image, resulting in a high score (yellow nodule) in the map with SI_out_ - SI_in_ values. The blue nodule seen in the fat fraction map indicates a low fat fraction inside the MMLn.

All the fatty hila of 40 NLns from 25 patients showed SI_in_>SI_out_. In the cortex of the 113 NLns from 40 patients, SI_out_>SI_in_ occurred in 41/113 cortices from 19/40 patients (47.5%). As to MMLns, SI_out_>S_in_ occurred in 8/8 cases (100%) (Fig. 3 and Fig. 4).

The fat fraction of MMLns was significantly lower than that of NLns (10% vs 89%, p<0.001). We elected not to measure the fat fraction of NLns cortex because the pixel ROI had been placed in a low-fat content area as a reference position, so all fat fractions in NLns cortex can be considered to be negligible.

## 4 Discussion

While US can be advantageous for the assessment of superficial lymph nodes (allowing a prompt measurement of both short and long axes, providing clues about node morphology and echogenicity that may suggest a benign or malignant nature, and serving as imaging guide for biopsy), MRI is usually superior for the evaluation of deep-seated lymph nodes. Moreover, the possibility to combine morphological information with quantitative data derived from multiple MRI sequences can improve the diagnostic performance of MRI in terms of lymph node characterization, especially when morphology alone (e.g., in case of smaller lymph nodes) cannot allow a reliable differentiation between normal and metastatic lymph nodes.

Our purpose was to explore the potential of the DIXON sequence for noninvasively distinguishing normal from metastatic lymph nodes in patients with skin melanoma. This sequence is performed using a dual-echo technique in one excitation, resulting in the generation of an out-of-phase and an in-phase series. On 3.0-Tesla MRI equipment, fat and water protons give their maximum contribution to SI at 2.4 ms (TE in-phase), whereas at 1.2 ms (TE out-of-phase) the signals of fat and water protons cancel one another, resulting in maximal signal loss in the same voxel of the out-of-phase series [14]. Our findings showed that in the hilum of NLns, SI_out_ was consistently lower than SI_in_ due to the drop-out effect generated by multiple water-fat interfaces inside the normal lymph node structure [15]. Conversely, in our NLns cohort a SI_out_>SI_in_ pattern was detected in the cell-rich cortex of 41/113 NLns, indicating that no or minimal fat might be present in NLns cortex.

In MMLns, metastatic involvement starts in the lymph node cortex and gradually extends to the hilum [16]. In our MMLns cohort, we reported a SI_out_>SI_in_ pattern in 8/8 MMLns, possibly due to the drop-in effect related to the paramagnetic properties of melanin inside melanoma metastatic cells [15], [17]. While further investigation is warranted to corroborate this hypothesis (by testing the SI behavior on the DIXON sequence of lymph node metastases from non-melanoma cancers), it can be presumed that SI_out_ - SI_in_ maps can be useful to highlight the drop-in effect. Maps will show a round-shaped drop-in effect in MMLns, and will demonstrate a peripheral, thin rim in those NLns cortices with drop-in effect, aiding in the differentiation between MMLns and NLns.

By summing up SI values from in- and out-of-phase series, it is possible to derive a water_only series, whereas a fat_only series can be generated by subtracting the same values from one another. In our patient sample, the fat fraction of MMLns was significantly lower compared to the hilum of NLns. This finding is consistent with previous studies, in which low fat fractions were detected in metastatic nodes from breast [18] and prostate [19] cancer. Of note, the low fat fraction of MMLns can make them far less subject to pixel shift than the fat-rich tissue in NLns, easing the detection of this biomarker.

At the border between viable tissue and fat, phase cancellation between the SI of water and fat protons results in a signal null in out-of-phase images, leading to a black boundary artifact due to chemical shift [20]. In NLns, the black boundary artifact delineates the outer side of cortex rim and the inner side between the cortex and fatty hilum. In contrast, in MMLns the black boundary artifact demarcates the outer nodal contour, as the fatty hilum is invaded by metastatic cells. In this context, the black boundary artifact seen on DIXON images can help differentiating NLns from MMLns, as a lymph node with a black boundary artifact outlining the cortex relative to the fatty hilum is supposed to be benign. In our cohort, we found 5 complete black boundary outlines in peripheral MMLns and 2 around mesenteric MMLns, all of which having a round shape. The black boundary artifact was incomplete in MMLns at the lung hilum and in the subcarinal region, because of boundary pixel contact with air and neighboring structures, such as the esophagus and the left atrium.

To our knowledge, this was the first attempt to create a drop-in map combined with a fat fraction map to facilitate MMLns detection. These two maps should incentivize the automation of MMLns detection in the future, contributing to a more timely diagnosis through the introduction of novel quantitative biomarkers. Furthermore, the interpretation of MR-WBI maps could increase the radiologists’ confidence in the differentiation between NLns and MMLns, possibly improving their diagnostic performance and their overall workflow.

In addition to MR-WBI, 18-fluoro-deoxyglucose (^18^F-FDG) PET/CT has an established role in the surveillance of patients with melanoma. Although ^18^F-FDG PET/CT has a sensitivity of 94% and specificity of 85% in the detection of melanoma recurrence, it can be hindered by false positive results (ranging from 7% to 14% in malignant melanoma, including MMLns) [21], [22]. In this setting, performing an MRI examination of the suspected metastatic site using the DIXON sequence might dictate a watch-and-wait approach at least in selected patients with positive ^18^F-FDG PET/CT imaging.

### 4.1 Limitations

The main limitation of our study was represented by the small sample size (especially in the MMLns group, N=8), with all patients having been recruited in a single center. This prevented us from performing a statistically meaningful calculation of the diagnostic accuracy, e.g., in terms of false-positive and false-negative rates of MMLns with SI_out_>SI_in_. Despite this [23], we believe that the combination of a SI_out_> SI_in_ pattern, low fat fraction and rounded shape can be considered as promising preliminary findings, which need to be corroborated on a larger patient sample collected on a multicenter basis.

Another limitation is that our findings may not be specific to MMLns, because the drop-in effect can also be seen in normal structures (such as the NLns cortex in 36% of our cases) and in case of iron deposition. Nonmetastatic lymph nodes can be the site of iron deposits due to iron metabolism disorders, as can happen e.g. in patients with hemosiderosis, chronic inflammation [7], and blood diseases [5], [6]. A low fat fraction and a rounded shape can also be encountered in lymph node metastases from other neoplasms, such as breast and prostate cancer [18], [19].

## 5 Conclusion

Early detection of disease relapse is the goal of MR-WBI surveillance in patients with malignant melanoma, creating an opportunity for curative surgery or successful systemic therapy [20]. As DWI/ADC images can only be moderately helpful for detecting small lymph node metastases, the combination of rounded shape, drop-in effect and low fat fraction on DIXON images can provide insights for differentiating NLns from MMLns, and may form the basis for an integrated, possibly automated assessment of disease recurrence. Further studies are warranted to corroborate our preliminary findings on larger patient cohorts.

### Data statement

The data that support the findings of this study are available on request from the corresponding author, [CB].

## Funding

This research received no specific grant from funding agencies in the public, commercial, or not-for-profit sectors.

## Ethical statement

The ethical committee of our hospital approved this study (ClinicalTrials.gov Identifier: NTC02907827). The study was conducted in accordance with relevant laws, international guidelines (e.g., the Declaration of Helsinki) and institutional ones. Informed consent was obtained and documented on paper.

## CRediT authorship contribution statement

**C. Brussaard**: Writing – review & editing, Writing – original draft, Visualization, Validation, Software, Project administration, Methodology, Investigation, Formal analysis, Data curation, Conceptualization. **F.E. Ramirez-Barbosa**: Visualization, Software, Methodology, Formal analysis. **L. Faggioni**: Writing – review & editing, Supervision, Investigation. **J. de Mey**: Visualization, Supervision. **B. Neyns**: Resources. Y. Jansen: Resources. **M. Vervoort**: Visualization, Software, Methodology, Formal analysis. **E. Neri**: Supervision. **D. Cioni**: Writing – review & editing. **I. Willekens**: Validation, Data curation.

## Declaration of Competing Interest

The authors have no conflicts of interest.
